# Treatment outcomes and safety of afatinib in advanced squamous cell lung cancer progressed after platinum‐based doublet chemotherapy and immunotherapy (SPACE study)

**DOI:** 10.1111/1759-7714.13880

**Published:** 2021-02-14

**Authors:** Seung Hyeun Lee, Cheol‐Kyu Park, Sung Yong Lee, Chang‐Min Choi

**Affiliations:** ^1^ Division of Pulmonary and Critical Care Medicine, Department of Internal Medicine, Kyung Hee University Medical Center Kyung Hee University College of Medicine Seoul South Korea; ^2^ Department of Internal Medicine Chonnam National University Hwasun Hospital Hwasun South Korea; ^3^ Division of Pulmonary, Allergy, and Critical Care Medicine, Department of Internal Medicine, Korea University Guro Hospital Korea University College of Medicine Seoul South Korea; ^4^ Department of Pulmonary and Critical Care Medicine, Asan Medical Center University of Ulsan College of Medicine Seoul South Korea; ^5^ Department of Oncology, Asan Medical Center University of Ulsan College of Medicine Seoul South Korea

**Keywords:** afatinib, genomic profiling, next‐generation sequencing, real‐world, squamous cell carcinoma

## Abstract

Afatinib is an *ErbB* family blocker approved for the treatment of epidermal growth factor receptor mutation‐positive nonsmall‐cell lung cancer. A pivotal trial demonstrated significant clinical benefits with manageable toxicity of afatinib as a second‐line treatment option in squamous cell carcinoma of the lung (SCC) which led to approval in >60 countries. However, these results were derived from a controlled study conducted in selected patients and are not necessarily representative of the real‐world use of this drug. In addition, data on afatinib use after immunotherapy in this clinical setting are lacking. The aim of this study is to evaluate the treatment outcomes and safety of afatinib as a second‐ or later‐line treatment for SCC and to identify potential predictive biomarkers. As a real‐world observational study, 130 eligible patients with advanced SCC, who progressed after platinum‐based chemo‐ and immunotherapy, will be enrolled. Treatment outcomes and safety data will be collected for both the retrospective and prospective cohorts, and molecular profiling using tissue and plasma will be performed for the prospective cohort. The primary endpoint is time to treatment failure, and the secondary endpoints are objective response rate, progression‐free survival, overall survival, and safety. Comparison of clinical outcomes with respect to the different programmed death‐ligand 1 expression and molecular characteristics will also be carried out. This study will provide additional evidence on the usefulness of afatinib as a subsequent treatment, as well as feasible molecular biomarkers to predict its efficacy in this clinical setting.

## Graphical abstract

This study will evaluate the treatment outcomes and safety of afatinib as a second‐ or later‐line treatment for SCC and identify potential predictive biomarkers. As a real‐world observational study, 130 eligible patients with advanced SCC, who progressed after platinum‐based chemo‐ and immunotherapy, will be enrolled. Treatment outcomes and safety data will be collected for both the retrospective and prospective cohorts, and molecular profiling using tissue and plasma will be performed for the prospective cohort.

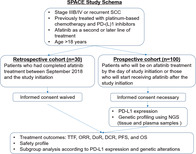

## INTRODUCTION

Squamous cell carcinoma of the lung (SCC) constitutes 30% of all lung cancers and is the second most common histological subtype of nonsmall‐cell lung cancer (NSCLC).^1^, ^2^ As with the majority of patients with lung cancer, approximately two‐thirds of patients with SCC are diagnosed at an advanced stage.^1^ While molecularly targeted therapy has revolutionized the treatment of adenocarcinoma with genetic alterations, including the epidermal growth factor receptor (EGFR) and anaplastic lymphoma kinase, treatment options for SCC are relatively limited compared to those of adenocarcinoma.^3^ Although platinum‐based chemotherapy has been used as a first‐line treatment, its clinical outcomes are still modest.^4^, ^5^

Recently, immune checkpoint inhibitors (ICIs), including pembrolizumab, nivolumab, atezolizumab, and the vascular endothelial growth factor receptor‐2 antibody ramucirumab (in combination with docetaxel), have been approved as new second‐line treatments for SCC.^5^, ^6^ Furthermore, pembrolizumab monotherapy for high programmed death‐ligand 1 (PD‐L1) expressors and taxane‐based chemotherapy combined with pembrolizumab regardless of PD‐L1 expression levels have been approved as novel first‐line treatments.^6^ However, overall clinical outcomes are still inferior compared to those for adenocarcinoma even with similar treatment strategies,^4^, ^7^, ^8^, ^9^ highlighting the unmet clinical need in this patient population.

SCCs are genetically complex and characterized by high mutation rates.^3^ Comprehensive molecular profiling has revealed that these cancers harbor numerous genomic and epigenomic alterations, with a reported mean of 360 exonic mutations, 165 rearrangements, and 323 segments of copy number alterations per tumor.^3^, ^10^ The Cancer Genome Atlas Project compared SCC tissue samples to normal pulmonary tissue to identify potential actionable mutations, and 11 common genomic abnormalities were observed, including those touching tumor protein 53, cyclin‐dependent kinase inhibitor 2A (*CDKN2A*), phosphatase and tensin homolog (*PTEN*), and *PIK3CA*.^10^ The genetic complexity of SCC is largely attributable to tobacco smoking, which compromises treatment success. With such a complex genetic landscape and associated high immunogenicity, this tumor type has been targeted for immunotherapy and chemotherapy. However, the development of targeted agents has, so far, represented a significant challenge.^11^

Studies have suggested that SCC is strongly dependent on the *ErbB* family pathway; *ErbB1* (*EGFR*) is overexpressed in about 70% of SCCs, whereas *ErbB2* and *3* are overexpressed in 20–30% of cases. In addition, gene copy‐number alterations of *EGFR* were found in 7–10% of SCCs.^12^, ^13^, ^14^, ^15^, ^16^ Although *EGFR* mutations were less common, 21.6% of SCC patients had at least one *ErbB* mutation.^16^, ^17^, ^18^, ^19^ These data provide a biological rationale for *ErbB*‐targeted therapy in this histologic subtype. Afatinib, an irreversible *ErbB* inhibitor, can inactivate aberrant *ErbB*‐dependent signaling pathways and has been postulated to be effective in treating SCC.^20^, ^21^ In the LUX‐Lung 8 trial, afatinib (40 mg/day, *n* = 398) was compared to erlotinib (150 mg/day, *n* = 397) in stage IIIB/IV SCC patients who had received at least four cycles of chemotherapy with platinum doublet agents.^22^ Afatinib was better in terms of both progression‐free survival (PFS) and overall survival (OS) (PFS 2.4 vs. 1.9 months, hazard ratio [HR] 0.82, 95% confidence interval [CI] 0.68–1.00, *p* = 0.041; OS 7.9 vs. 6.8 months, HR 0.81, 95% CI 0.69–0.95, *p* = 0.0077). Although a 1.1‐month difference in OS in a head‐to‐head comparison can be clinically questionable, especially considering its translation into routine clinical practice, the survival rate at 12 (36% vs. 28%, *p* = 0.016) and 18 months (22% vs. 14%, *p* = 0.013) was significantly higher with afatinib, suggesting a prolonged benefit of this drug.^22^ Furthermore, the safety profiles were similar in both groups. Based on the significant improvements in PFS and OS along with a manageable safety profile, in April 2016 the US Food & Drug Administration (FDA) approved afatinib as a new oral treatment option for SCC that progressed after platinum‐based chemotherapy. A subsequent analysis, which assessed the association between clinical outcomes and *ErbB* family member gene alterations, showed that PFS (median 4.9 vs. 3.0 months, HR 0.62, 95% CI 0.37–1.02, *p* = 0.06) and OS (median 10.6 vs. 8.1 months, HR 0.75, 95% CI 0.47–1.17, *p* = 0.21) was numerically longer among those with *ErbB* mutation‐positive disease than among those without.^16^ Furthermore, the presence of *ErbB2* mutations was associated with more favorable PFS and OS following afatinib than erlotinib treatment.^16^ These data suggest that specific molecular characteristics can be biomarkers for afatinib treatment success in SCC, and genomic profiling may help identify patients with SCC who may derive additional benefits from treatment with this drug.

Although ICIs, either used alone or in combination with chemotherapy, are recommended as first‐ and second‐line treatments in SCC, clinical data on afatinib use after both platinum‐based chemotherapy and ICI failure are lacking. To broaden our understanding of afatinib use in SCC, we aim to assess its efficacy and safety as a second‐ or later‐line treatment in real‐world clinical practice and to identify potential biomarkers using molecular profiling data. Our results could provide further support for the use of afatinib as a treatment option in patients who experience progression after both chemotherapy and immunotherapy. In addition, molecular biomarkers could help us predict response and select patients who will show favorable outcomes before afatinib use, which may facilitate “personalized medicine” in this clinical setting.

## PATIENTS AND METHODS

### Study design

**FIGURE 1:**
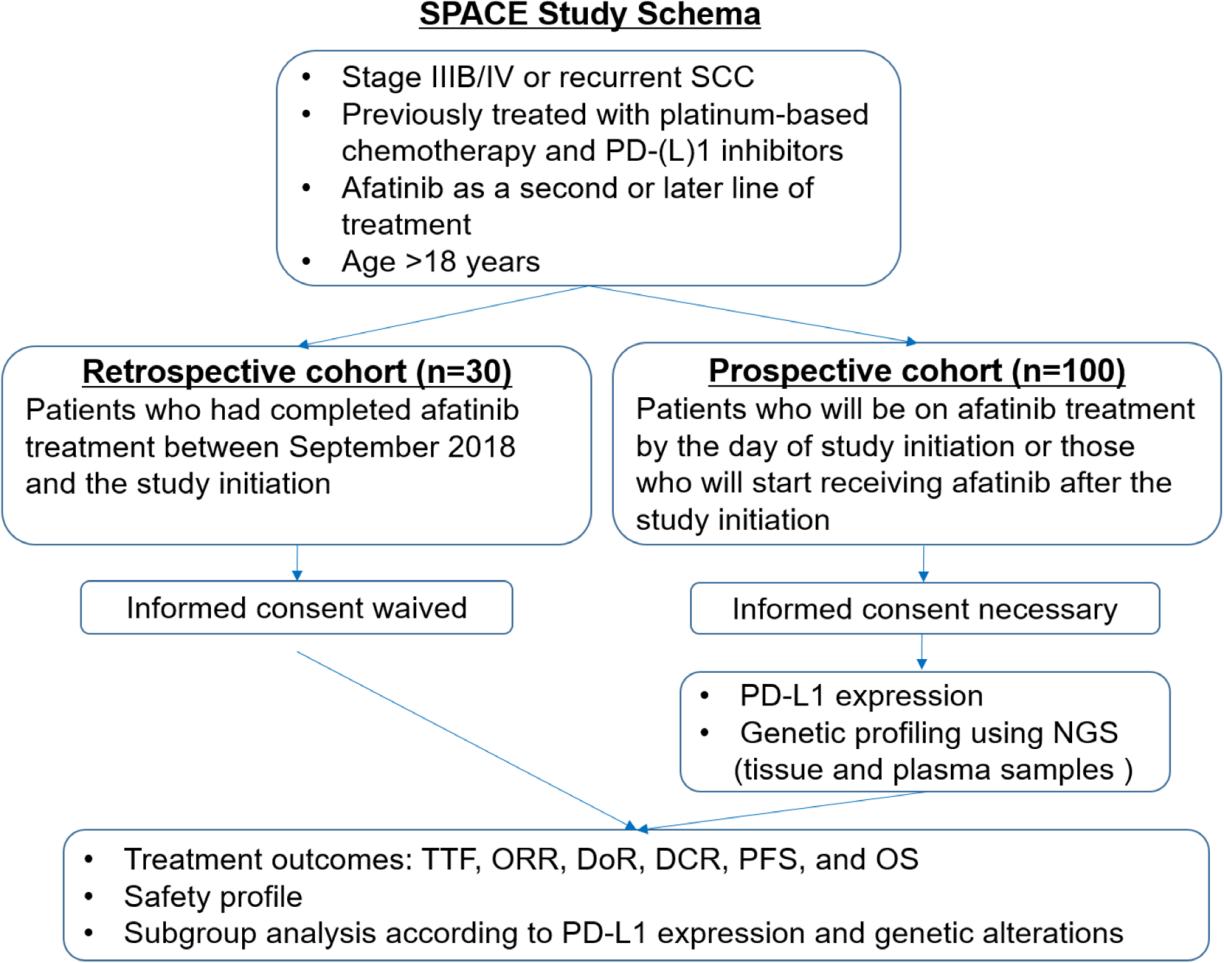
Study flowchart. DCR, disease control rate; DoR, duration of response; NGS, next‐generation sequencing; ORR, objective response rate; OS, overall survival; PD‐(L) 1, programmed death‐(ligand) 1; PFS, progression‐free survival; SCC, squamous cell carcinoma of the lung; TTF, time to treatment failure

The SPACE study is a retrospective and prospective, observational, multicenter study. Figure 1 shows the flow chart for this study. Our aim is to evaluate the treatment outcomes and safety of afatinib in patients with advanced SCC who progressed after first‐line platinum‐based chemotherapy plus immunotherapy or platinum‐based chemotherapy followed by immunotherapy. We aim to recruit 130 patients, for retrospective and prospective cohorts of 30 and 100 patients, respectively, from four institutes in South Korea from January 2021 to December 2021. The observational period will be 1 year from the time of final registration. This is not a powered study. The minimum sample size has been set at 130 eligible patients to provide meaningful results.

The primary endpoint of this study is the time to treatment failure (TTF), defined as the time from the start of afatinib treatment to the time of treatment discontinuation for any reason, including disease progression, treatment toxicity, and death. The secondary endpoints are the objective response rate (ORR), duration of response (DoR), disease control rate (DCR), PFS, OS, and safety profiles. In addition to these, the difference in treatment outcome parameters with respect to PD‐L1 expression and genetic alterations will be analyzed. This protocol has been approved by the Medical Ethics Committee of the Kyung Hee University Medical Center, Seoul, South Korea, and all participating institutes. As this study involves human participants, all procedures will be performed in accordance with the ethical standards of the Institutional and National Research Committee, and the 1964 Declaration of Helsinki and its later amendments or comparable ethical standards. This study is subject to the supervision and management of the ethics committees of all participating institutes. This study is registered in the Clinical Research Information Service (CRIS, https://cris.nih.go.kr, registration number KCT0005712), the official registration site for clinical trials powered by the Korea Disease Control and Prevention Agency.

### Eligibility criteria

The inclusion criteria are as follows: (a) histologically confirmed as advanced, metastatic, or recurrent SCC; (b) history of first‐line platinum‐based doublet chemotherapy plus immunotherapy or first‐line platinum‐based chemotherapy followed by subsequent immunotherapy; (c) receiving afatinib as a second‐ or later‐line treatment; (d) age >18 years; and (e) provision of written informed consent. Patients who were treated with other EGFR‐tyrosine kinase inhibitors will be excluded.

Afatinib was approved by the Korean FDA in September 2018 for the treatment of SCC. Patients who had completed treatment with afatinib before the initiation of the study will be assigned to the retrospective cohort. Patients who will be on afatinib treatment by the day of initiation of the study or those who will start receiving this drug after the initiation of the study will be assigned to the prospective cohort. Written informed consent will be waived for patients in the retrospective cohort.

### Treatment plan

As per routine practice, patients will be treated with afatinib 40, 30, or 20 mg tablets once daily, as indicated in the approved labels of the drug. Dose reduction will be allowed based on patient tolerability. Drug administration will be continued until disease progression or significant side effects occur. Based on the recommendations of the current guideline,^23^ “beyond‐progression” use is permitted in case of oligo‐progression with appropriate local treatment.

### Data collection

Clinical parameters, survival data, and baseline PD‐L1 expression level data, obtained using 22C3 or SP263 assays, will be collected from medical chart reviews. Tumor response will be assessed using computed tomography which is to be performed at least 8‐week interval of during treatment and evaluated according to the Response Evaluation Criteria in Solid Tumors (RECIST) 1.1.^24^ Positron emission tomography or brain magnetic resonance imaging will be used, when necessary, as per routine practice. For patients in the prospective cohort, molecular profiling using targeted next‐generation sequencing (NGS) will be performed on tumor tissue obtained at diagnosis and plasma samples obtained before the initiation of afatinib. For efficacy evaluation, treatment outcome parameters, including TTF, ORR, DoR, PFS, and OS, will be calculated. Subgroup analysis will be performed with respect to PD‐L1 expression levels and genetic alterations. Possible treatment‐related adverse events (TRAE) during afatinib treatment, including stomatitis, skin reaction, paronychia, diarrhea, and elevated liver enzymes, will be assessed based on the Common Terminology Criteria for Adverse Events (CTCAE Version 5.0).^25^ In addition, information on immune‐related adverse events (irAE), including pneumonitis, colitis, and hepatitis, will be collected to assess possible association between irAE and TRAE.

## MOLECULAR PROFILING USING NGS

Tumor DNA will be extracted from sections (40‐μm thick) of formalin‐fixed, paraffin‐embedded (FFPE) tumor tissue using the QIAamp DNA FFPE Tissue Kit (Qiagen). Plasma will be isolated from 8 ml of whole blood using density gradient centrifugation in Ficoll‐Paque™ PLUS (GE Healthcare). Cell‐free DNA (cfDNA) will be extracted from isolated plasma samples using the QIAamp Circulating Nucleic Acid Kit (Qiagen). The quantity and quality of tumor DNA and cfDNA will be assessed using a 4150 TapeStation System (Agilent) and a Qubit 3.0 fluorometer (Thermo Fisher Scientific), respectively.

Targeted NGS will be performed using Cancer‐PRIME™ (Clinomics Inc.). This panel was designed to characterize single‐nucleotide variants (SNVs), insertion/deletion, and copy number variations in 51 actionable genes. Candidate genes are considered to be included in the cancer panel if they are associated with FDA‐approved therapies or reported clinical trials. The list of genes in this panel is shown in Table 1. The NGS‐based clinical cancer gene assay has been previously published and the assay performance has been validated.^26^, ^27^, ^28^ In brief, 1 μl of purified DNA from each sample will be used to analyze its quality and size using a Bioanalyzer system (Agilent). In addition, DNA concentration will be assessed using the dsDNA BR assay using a Qubit fluorometer (Thermo‐Fisher Scientific). To filter out potential sequencing backgrounds, we will exclude variants that are detected in DNA samples from 30 healthy individuals. Common SNVs obtained from the whole‐genome sequencing data of 50 healthy unrelated Korean individuals will also be excluded.^29^

**TABLE 1:** List of the 51 actionable genes in the cancer‐PRIME™ next‐generation sequencing panel

ABL1	BTK	ERBB3	GNA11	MET	PPARG	TP53
AKT1	CCND1	ERBB4	GNAQ	MTOR	PTCH1	TSC1
ALK	CDK4	ESR1	HRAS	MYCN	PTEN	TSC2
AR	CDK6	FGFR1	IDH1	NOTCH1	RAF1	
BAP1	CDKN2A	FGFR2	IDH2	NRAS	RET	
BRAF	DDR2	FGFR3	KIT	PDGFRA	ROS1	
BRCA1	EGFR	FGFR4	KRAS	PD‐L1	SMO	
BRCA2	ERBB2	FLT3	MAP2K1	PIK3CA	STK11	

## STATISTICAL ANALYSIS

All of the time‐to‐event data on treatment outcome parameters will be estimated using the Kaplan–Meier method. The median value and a two‐sided 95% CI will be described. Univariate and multivariate Cox regression analyses will be performed to estimate the potential clinical parameters or molecular subtypes associated with treatment outcomes.

## DISCLOSURES

The authors have no conflicts of interest to declare.
